# Cybersecurity maturity assessment framework for higher education institutions in Saudi Arabia

**DOI:** 10.7717/peerj-cs.703

**Published:** 2021-09-09

**Authors:** Iman Almomani, Mohanned Ahmed, Leandros Maglaras

**Affiliations:** 1 Security Engineering Lab, Prince Sultan University, Riyadh, Saudi Arabia; 2 Computer Science Department, The University of Jordan, Amman, Jordan; 3 School of Computer Science and Informatics, De Montfort University Leicester, Leicester, United Kingdom

**Keywords:** Saudi Arabia, Cybersecurity, Maturity assessment, Audit tool, ISO27001, CITC, NCA, ECC, CRF, GDPR, COVID-19, Higher education

## Abstract

The Saudi Arabia government has proposed different frameworks such as the CITC’s Cybersecurity Regulatory Framework (CRF) and the NCA’s Essential Cybersecurity Controls (ECC) to ensure data and infrastructure security in all IT-based systems. However, these frameworks lack a practical, published mechanism that continuously assesses the organizations’ security level, especially in HEI (Higher Education Institutions) systems. This paper proposes a Cybersecurity Maturity Assessment Framework (SCMAF) for HEIs in Saudi Arabia. SCMAF is a comprehensive, customized security maturity assessment framework for Saudi organizations aligned with local and international security standards. The framework can be used as a self-assessment method to establish the security level and highlight the weaknesses and mitigation plans that need to be implemented. SCMAF is a mapping and codification model for all regulations that the Saudi organizations must comply with. The framework uses different levels of maturity against which the security performance of each organization can be measured. SCMAF is implemented as a lightweight assessment tool that could be provided online through a web-based service or offline by downloading the tool to ensure the organizations’ data privacy. Organizations that apply this framework can assess the security level of their systems, conduct a gap analysis and create a mitigation plan. The assessment results are communicated to the organization using visual score charts per security requirement per level attached with an evaluation report.

## Introduction

Many organizations worldwide aim to enable digital transformation successfully. Digital Infrastructure Development is one of the main targets of Saudi Arabia Vision 2030 (https://www.vision2030.gov.sa/en). Any digital transformation success depends heavily on achieving the security of both data and infrastructure, whether in the public or private sectors. Securing IT systems in all Saudi organizations needs special attention to ensure smooth and secure digital transformation (Iwendi et al., 2020; Sagar, Jhaveri & Borrego, 2020).

Such transformation is more stressed after the COVID-19 pandemic where almost all life activities were forced to become digital. Almost all sectors were influenced by this sudden change, including the education sector (Alshehri et al., 2020; Iivari, Sharma & Ventä-Olkkonen, 2020; Mhlanga & Moloi, 2020). Moreover, Ali (2020) stated that online and remote learning is now essential because of lockdown due to the Coronavirus pandemic. However, many challenges appeared when trying to digitize the learning process, as many Higher Education Institutions (HEIs) were not ready to use E-learning systems (Kaur, 2020). Therefore, through this pandemic, we learned that having a remote learning infrastructure is crucial (Alshehri et al., 2020). However, in HEIs, there is a lack of technical skills needed in their systems (Ali, 2020; Kaur, 2020), including cybersecurity skills. Therefore, it is crucial to start improving the education applications and how people are using them (Alshehri et al., 2020; Ali, 2020). In Saudi Arabia, E-learning is active in prominent higher education institutions such as King Saud University and King Abdul-Aziz University before the pandemic. However, after the pandemic transforming to mobile/eLearning education was not an option. All educational Institutions switched to online teaching, as directed by the ministry of education (MoE). Yet, there were many challenges to apply tutoring and assessments digitally (Hassounah, Raheel & Alhefzi, 2020; Henriette, Feki & Boughzala, 2016; Atawneh et al., 2020) and to secure the e-leaning services.

There are existing frameworks and standards defined at the local and international levels that provide cybersecurity regulatory guidelines for organizations. However, there is still a lack in (a) studying the current Saudi Arabia cybersecurity frameworks in general and in the context of higher education in specific, (b) proposing a cybersecurity maturity model that is comprehensive enough to cover the approved frameworks by the Saudi government including the Essential Cybersecurity Controls (ECC) introduced by the National Cybersecurity Authority (NCA) and the Cybersecurity Regulatory Framework (CRF) developed by the Communications and Information Technology Commission (CITC) (c) providing tools to assess the cybersecurity maturity of the organizations.

The importance of offering the services of the cybersecurity maturity models in terms of well-developed tools is that the organizations can apply them to perform self-assessment in order to (a) check their cybersecurity maturity status (b) discover their weaknesses and set a clear plan for improving their security level and protecting their systems and services from different security attacks (c) prepare the organization to become certified by well-known security agencies like ISO; such self-assessment could be a preliminary step toward achieving that.

**Figure 1:**
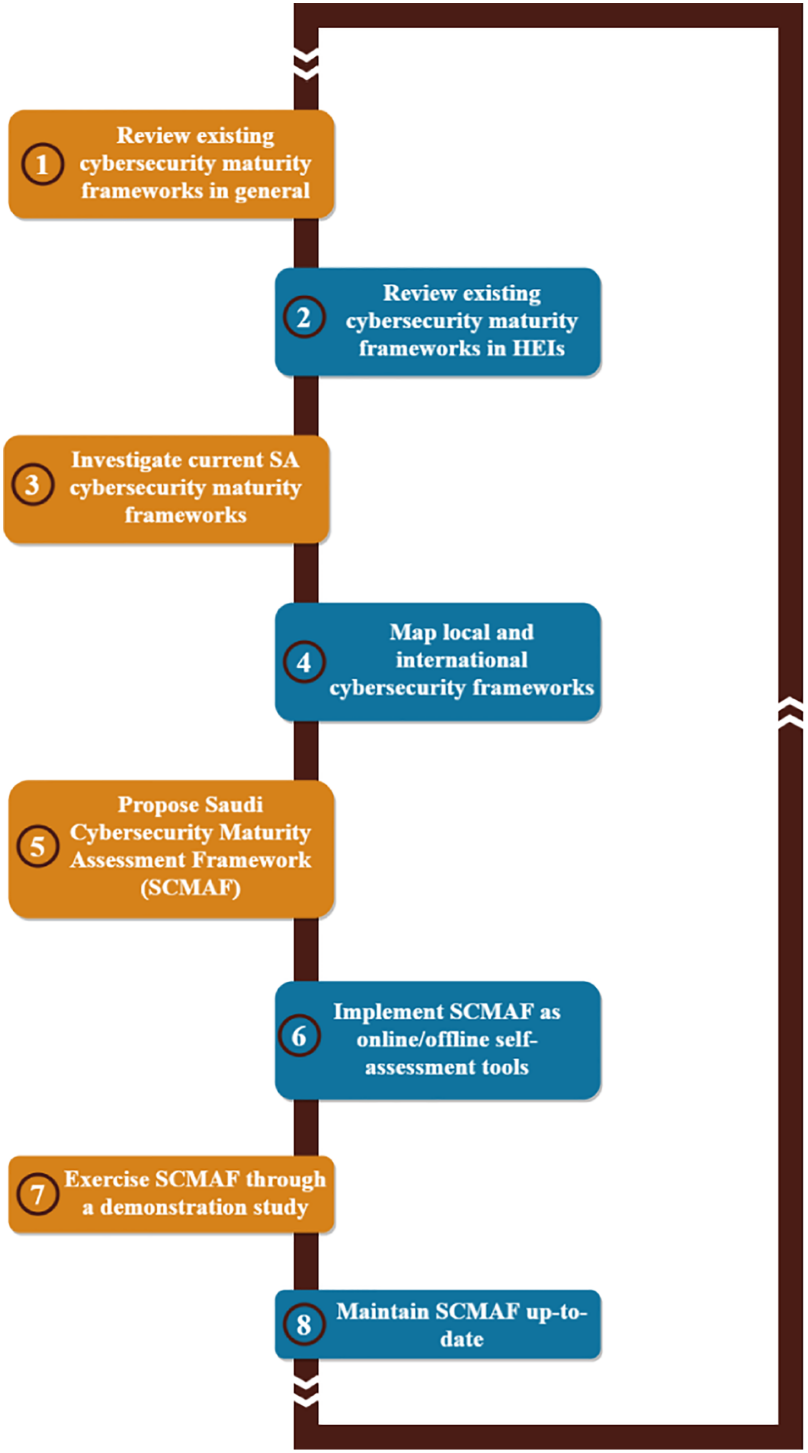
Research methodology.

Therefore, this research proposes a comprehensive, customized cybersecurity maturity assessment framework (SCMAF) for HEIs in Saudi Arabia. SCMAF has considered both the Saudi local security standards in addition to the international standards. SCMAF is offered to organizations in the form of an online web-based tool or offline standalone tool to assess their systems’ security maturity levels in a convenient way that ensures their data secrecy and integrity. The results of the assessment will be communicated in terms of scores per security requirement attached with a full report. Overall, the methodology followed to build SCMAF is shown in Fig. 1 and summarized as follows:

- Review existing cybersecurity maturity frameworks in general.
- Review existing cybersecurity maturity frameworks in HEIs.
- Investigate current SA cybersecurity maturity frameworks.
- Map local and international cybersecurity frameworks.
- Propose Saudi Cybersecurity Maturity Assessment Framework (SCMAF).
- Implement SCMAF as online/offline self-assessment tools.
- Exercise SCMAF through a demonstration study.
- Maintain SCMAF up-to-date.

The rest of the paper is organized as follows. ‘Related Work’ presents recent related works and current cybersecurity standards. ‘Proposed Cybersecurity Maturity Assessment Framework’ introduces the proposed Saudi Cybersecurity Maturity Assessment Framework (SCMAF). ‘Conclusions’ shows the implementation of SCMAF and discusses its contributions. ‘LImitations And Future Works’ draws conclusions and suggests possible future work.

## Related work

### Importance of HEIs security

Digitizing the educational services makes the HEIs more targeted by cybersecurity attacks, like any other organization. However, HEIs lack the security awareness needed to handle these security issues (Hina & Dominic, 2018). Therefore, they could use systems such as m-learning systems with security risks (Badwelan, Drew & Bahaddad, 2016). This could happen because there is neither standards nor guidelines to be applied or forced in HEIs. Moreover, most organizations do not monitor their users’ misbehavior (Hina & Dominic, 2018). Since not all IT departments in higher education institutions/universities have the same experience and understanding of how important cybersecurity is, they do not treat it as a continuous process. Therefore, they are relying on the security of well-known systems, which is not always enough. For example, Michigan University (MU) data was breached, which caused private information to be stolen (Al-Serhani et al., 2018). Another data leakage in HEIs was occurred at the University of Texas, Austin. In this incident, nearly 200,000 students’ electronic records were accessed illegally (Marks, 2007). The risk of security vulnerabilities rises because of the lack of technical knowledge and social awareness of these threats (Makupi & Masese, 2019).

Hence, the higher education institutions and universities should learn lessons from the previous hacking scenarios in the HEI services and be always ready to defend against all possible attacks that might target their digital, computer-based systems and start improving the security at all the levels: technical, physical, and administrative (Al-Serhani et al., 2018; Makupi & Masese, 2019). This is important because if the platforms used in HEIs are not secure enough, the copyright of learning materials might not be secure as well (Alrasheedi & Capretz, 2013). Furthermore, cybersecurity experts find that improving cybersecurity education helps in preventing security attacks in other critical sectors such as health. This is because the education sector is the entry point to any professional life (Ajmi et al., 2019).

In Saudi Arabia, the government is investing a lot in improving information technology sectors, especially education. However, challenges arise to handle the security threats that target these organizations (Alotaibi & Elnaim, 2020).

### Related cybersecurity maturity models and standards

Many methodologies have been proposed to improve cybersecurity practices within organizations. There are different types of methods used to do such proposals. For instance, Gerl et al. (2021), stated that to improve organizations’ security, framework standards such as COBIT can be applied. Proença & Borbinha (2018) presented a maturity model based on ISO/IEC 27001 to improve information security management systems. Another method, followed by Altameem (2013) which uses qualitative research to propose different factors to improve the security of systems.

Differently, various cybersecurity maturity models were developed according to the needs of organizations. Nowadays, the most common cybersecurity maturity models are created using different national and international standards such as ISO/IEC 27001, the National Institute of Standards and Technology (NIST), and European & American standards for cybersecurity. Moreover, ISO/IEC 27001 was created based on ISO/IEC 17799 and the British Standard BS7799. Its purpose is to improve and maintain the Information Security Management System (ISMS) by providing related requirements. Also, ISO/IEC 27001 considers ISMS as a component of the management system. ISMS takes care of establishing, implementing, operating, monitoring, reviewing, maintaining, and improving information security.

Even though the current used maturity models provide a way for assessing the security maturity level in different systems and organizations, it is not easy to create cybersecurity models and establish mechanisms for protecting cyberspace because both cyberspace & cybersecurity definitions and scopes are still not well defined. Additionally, existing maturity models establish basic compliance models and not flexible security models responsive to the new emerging security threats. Moreover, these models should enable users of various perspectives from all levels, such as practitioners, security experts, and management. This helps in measuring the overall security level of the organization/system and addressing its weaknesses. Finally, current maturity models use qualitative metrics or processes without considering quantitative metrics as an essential aspect for security assessment (Aliyu et al., 2020). Almuhammadi & Alsaleh (2017) presented a maturity model based on NIST Cyber Security Framework (CSF). The proposed maturity model compares NIST CSF to other security-related standards and frameworks such as COBIT and ISO/IEC 27001 (Almuhammadi & Alsaleh, 2017).

For higher education institutions, different maturity models were presented. Makupi & Masese (2019) introduced a model based on ISO 27001 to know the maturity level of cybersecurity in universities using clauses and compliance levels related to higher education institutions. Another model was proposed by Bass (2011) called ICT Maturity Model. The model consists of eight levels derived from different documents and results of the chosen higher education institutions and schools’ analysis. Yaokumah & Dawson (2019) used ISO/IEC 21827 maturity model for measuring the security controls in Ghanaian higher educational institutions. Suwito et al. (2016) created a maturity assessment model for Indonesian higher education institutions by combining different standards and models such as COBIT® 4.1, ITIL v3, and ISO/IEC 27001. Hung, Hwang & Liu (2013) did a study to improve Taiwanese universities’ Information Security Governance (ISG) maturity by doing a questionnaire survey. Then, the ISG maturity model was built by extracting the relevant features. Ismail et al. (2010) designed information security framework specific for higher education institutions in Malaysia. The authors in Aliyu et al. (2020), presented a web-based maturity model as an assessment tool for cybersecurity in higher education institutions in the UK. The model is called Holistic Cybersecurity Maturity Assessment Framework (HCYMAF) that covers privacy and cybersecurity regulations in higher education institutions. HCYMAF basic idea was to map the general security requirements derived from cybersecurity best practices (*e.g*., NIST Framework) with certification tools and regulations such as PCI DSS, GDPR, and DSPT. Bolanio et al. (2021) used ISO 27033 standard to help in improving network security in higher education institutions. Another approach proposed by Aedah & Hoga (2020) applied ISO 27001:2013 standard as a way to measure the maturity level of information system security practices in HEIs.

### Saudi Arabia security, maturity frameworks, and standards

In Saudi Arabia (SA), securing an organization is very important because of the high percentage of attacks compared to other countries. SA was targeted by many attacks, such as the Triton attack at ARAMCO and the Interior Ministry attack, called TASNEE (Ajmi et al., 2019). For HEIs, unlike Europe and North America, when it comes to universities, there might be a shortage of IT staff in KSA universities with immature ICT infrastructure (Alharthi et al., 2017). Chan & Mubarak (2012) did a study on the higher education sector and found that employees lack information security awareness. Therefore, security issues should be taken seriously when developing an infrastructure for universities (Ahmed, Buragga & Ramani, 2011). To face such issues, the Saudi Arabia government introduced multiple laws two of them are: e-transactions law, to regulate online transactions, and anti-cybercrime law, to limit the crimes of abusing IT, computers, and Internet (Ministry of Communications and Information Technology of Saudi Arabia, 2010; Ministry of Communications and Information Technology of Saudi Arabia, 2007). The National Cybersecurity Authority (NCA) developed Essential Cybersecurity Controls (ECC) based on national and international standards and laws to help organizations follow the best cybersecurity practices (National Cybersecurity Authority of Saudi Arabia, 2018). Based on these controls and other international standards, the Communications and Information Technology Commission (CITC) created a framework for the ICT sector entitled Cybersecurity Regulatory Framework (CRF) (Communications and Information Technology Commission of Saudi Arabia (CITC), 2019) which will be discussed in the following section. Moreover, a security policies framework for government agencies in need of such standards in cybersecurity were formerly developed (Communications and Information Technology Commission of Saudi Arabia (CITC), 2011). To regulate and secure financial services, the Saudi Arabian Monetary Authority (SAMA) developed two frameworks. First, Cyber Security Framework to identify and handle the risks that face financial transactions (Saudi Arabian Monetary Authority (SAMA), 2017b). Second, Business Continuity Management (BCM) framework for organizations to ensure the availability and continuity of the services (Saudi Arabian Monetary Authority (SAMA), 2017a). For higher education, National eLearning Center introduced a guide to general quality control standards such as data privacy standard (National eLearning Center KSA, 2020). In academic literature, Alnatheer & Nelson (2009), proposed a conceptual framework to identify factors that aid in improving information security culture in Saudi Arabia. Another work was done to improve online retailing sector by (AlGhamdi, Drew & Alhussain, 2012). In this paper, the authors presented a five-part conceptual model to enable trust in online retailing, and one of the parts was improving the security of consumers in Saudi Arabia (AlGhamdi, Drew & Alhussain, 2012). A security legal framework was built for Saudi Arabia by using institutional theory (Singh & Alshammari, 2020). The authors (Al Hamed & Alenezi, 2016) developed a maturity model to measure the capability of business continuity management and disaster recovery (BCM/DR) for IT companies in Saudi Arabia. The objective of the model was to compare BCM/DR to CITC practices along with the security standard ISO 22301:2012 (Al Hamed & Alenezi, 2016).

### Saudi Arabia HEIs frameworks

To improve the security in Saudi Arabia’s higher education institutions, some models proposed in the literature and will be highlighted in this section. The authors of Ajmi et al. (2019) proposed a holistic cybersecurity model to create an effective collective measure that incorporates three sub-models (Educational, Healthcare, and Commerce) that target Small and Medium Enterprises (SMEs). These models are linked together such that one type of SMEs can share its expertise and intelligence to fulfill the needs of the other types of SMEs (Ajmi et al., 2019). Aziz & Shahzad (2015) proposed factors to measure the quality of Information Technology Enabled Services (ITES) in higher education institutions. One of these factors was related to security features implemented in ITES. However, this model was simple and did not consider Saudi Arabia’s regulations and recognized standards.

Table 1 shows a comparative analysis among the related, existing cybersecurity maturity frameworks in terms of their purposes, the scope they cover, whether generic or HEI-specific, the standards they followed, and if their coverages were national or international. In the context of SA HEIs, the table highlights (a) if the proposed Saudi maturity assessment framework has followed the critical SA cybersecurity standards, including CRF and ECC, and (b) if the framework is implemented as a service (IaS). This allows the institution to self-measure its cybersecurity maturity level against Saudi and international standards using a lightweight and user-friendly tool. For examples, some of the proposed frameworks were general such as (Proença & Borbinha, 2018; Almuhammadi & Alsaleh, 2017). Others were HEI-specific like (Makupi & Masese, 2019; Bass, 2011; Yaokumah & Dawson, 2019; Aliyu et al., 2020) that based mainly on international standards. In Saudi Arabia, different approaches were presented to improve cybersecurity in general and HEIs in particular (Alnatheer & Nelson, 2009; Singh & Alshammari, 2020; Ajmi et al., 2019). The rest of comparisons are shown in Table 1. Even though there are attempts to propose maturity models for HEIs, these models neither fit the current SA regulatory standards and policies nor complete enough to provide practical maturity models. For example, there are no existing models that have considered CRF or/and ECC. Moreover, the proposed models were not implemented in terms of software-based services. Additionally, the current models are either international-based only or local-based only but not an integration of both. Therefore, this research incorporates the current cybersecurity regulations and standards in SA and internationally to propose a practical model to assess the cybersecurity maturity in HEIs’ systems in specific.

**Table 1:** Comparative analysis among the related cybersecurity frameworks.

Related work	Framework purpose	Scope	Followed standards	Coverage	CRF/ECC?	IaS?
Gerl et al. (2021)	Choose COBIT to guarantee completeness in IT governance in German HEIs	HEIs	• COBIT	International	–	–
Proença & Borbinha (2018)	Present a maturity model to improve info security management systems	General	• ISO27001	International	–	–
Singh & Alshammari, 2020	Build a security legal framework for SA using institutional theory	General	• Saudi Laws	National	–	–
Almuhammadi & Alsaleh, 2017	Propose a maturity model based on NIST Framework (CSF)	General	• NIST CSF	International	–	–
			• ISO27001			
			• ISF			
			• COBIT 5			
Makupi & Masese, 2019	Find cybersecurity maturity level in universities using clauses and compliance levels related to HIEs	HEIs	• ISO27001	International	–	–
Bass (2011)	Present ICT maturity model of the chosen HEs in Ethiopia and schools’ analysis	HEIs	None	International	–	–
Yaokumah & Dawson, 2019	Measure the security controls in Ghanaian HEIs	HEIs	• ISO21827	International	–	–
Suwito et al. (2016)	Propose a maturity assessment model for Indonesian HEIs	HEIs	• COBIT 4.1	International	–	–
			• ITIL v3			
			• ISO27001			
Hung, Hwang & Liu (2013)	Propose an improvement to Taiwanese universities’ Information Security Governance (ISG) maturity	HEIs	None	International	–	–
Ismail et al. (2010)	Design an information security framework specific for HEIs in Malaysia	HEIs	• COBIT	International	–	–
			• ISO27001			
Aliyu et al. (2020)	Propose HCYMAF to assess the cybersecurity in HEIs in the UK	HEIs	• NIST	International	–	✓
			• PCI DSS			
			• GDPR			
			• DSPT			
Bolanio et al. (2021)	Propose an improvement to the network security in HEIs	HEIs	• ISO27033	International	–	–
Aedah & Hoga, 2020	Measure the maturity level of info system security practices in Indonesian HEIs	HEIs	• ISO27001	International	–	–
Ajmi et al. (2019)	Incorporates three sub-models to measure cybersecurity of SMEs in SA	Education, Healthcare, and Commerce	• NIST	National	–	–
Al Hamed & Alenezi (2016)	Measure the capability of business continuity management and disaster recovery (BCM/DR) for IT companies in SA	General	• ISO22301	National	–	–
Aziz & Shahzad, 2015	Measure the quality of Info Technology Enabled Services (ITES) in Saudi’s HEIs	HEIs	None	National	–	–
Altameem (2013)	Propose different factors to improve the security of HEI systems in Saudi Arabia	HEIs	None	National	–	–
AlGhamdi, Drew & Alhussain (2012)	Improve the security of consumers in Saudi Arabia	Online Retailing Sector	None	National	–	–
Alnatheer & Nelson, 2009	Identify factors to improve info security culture in SA	General	None	National	–	–
Proposed SCMAF	Propose a comprehensive cybersecurity maturity assessment framework for HEI in SA	HEIs	• SA’s CRF	Both	✓	✓
			• SA’s ECC			
			• NIST			
			• PCI DSS			
			• GDPR			
			• DSPT			

## Proposed cybersecurity maturity assessment framework

Special attention is given to achieving security in all IT-based systems, especially after the digital transformation. In Saudi Arabia, different local cybersecurity regularly models are provided inspired by intentional security standards. However, there is no straightforward, automated process that can support organizations, especially in the HEI, to check their security maturity toward these standards, identify their weaknesses, and start preparing their improvement plans.

The automated process needs to be implemented through an easy, user-friendly software that allows the stakeholders to quickly answer the questions related to the achievements of specific security requirements and controls and provide them with a maturity score and a compliance report.

The primary motivation of this research is to develop a comprehensive cybersecurity maturity assessment model that considers the critical approved Saudi standards and international standards. Then allow the organization to self-assess its IT-based systems using an online or offline tool to measure their cybersecurity maturity level and act accordingly.

Therefore, this section presents the proposed Saudi cybersecurity maturity assessment framework (SCMAF). This framework aims to be comprehensive and includes both the international and the local Saudi cybersecurity standards. Therefore, the existing Saudi security-related standards are deeply studied, including ECC and the CRF as will be explained in the following subsections. Moreover, recent international maturity assessment framework related to HEIs is also considered and investigated. Afterwards, all these standards are mapped and enriched to introduce a comprehensive, customized cybersecurity maturity assessment model for Saudi HEIs. This model is offered as a self-assessment tool that can be applied by the institution itself to measure its cybersecurity maturity level and then acts accordingly.

### Essential cybersecurity controls (ECC)

**Figure 2:**
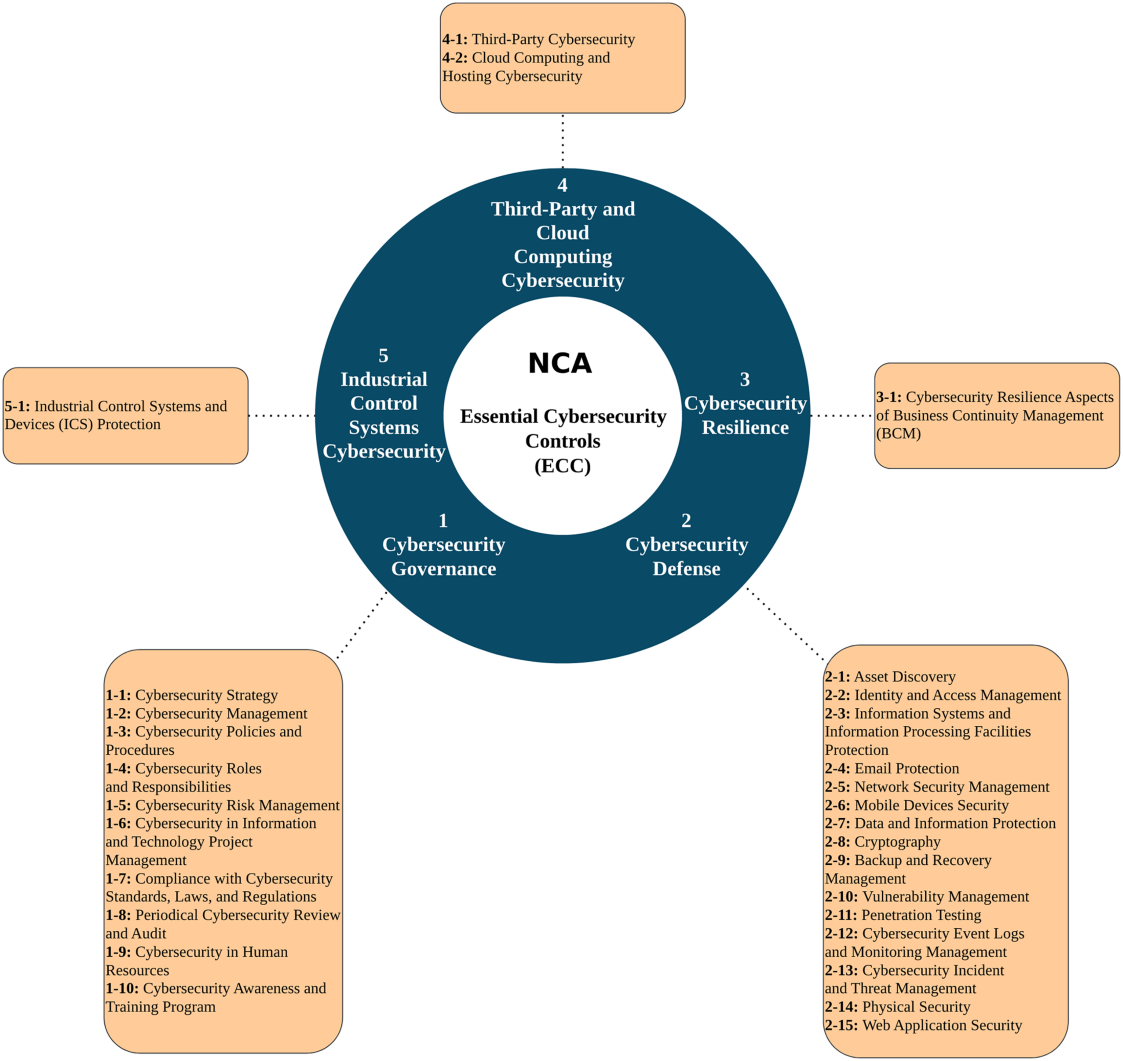
Essential Cybersecurity Controls (ECC).

In 2018, The National Cybersecurity Authority (NCA) introduced Essential Cybersecurity Controls (ECC) to ensure organizations’ minimum cybersecurity requirements in Saudi Arabia in both public and private sectors. ECC’s main objective is to safeguard and force confidentiality, integrity, and availability of the organization’s information and technology assets. Moreover, ECC is used by the government as a compliance assessment method. ECC was built based on national and international cybersecurity frameworks and standards along with KSA national laws. As shown in Fig. 2, ECC covers five main domains, which are Cybersecurity Governance, Cybersecurity Defense, Cybersecurity Resilience, Third-Party & Cloud Computing Cybersecurity, and Industrial Control Systems Cybersecurity. Under these domains, there are 29 sub-domains with 114 security controls (National Cybersecurity Authority of Saudi Arabia, 2018).

### Cybersecurity regulatory framework (CRF)

To increase the maturity level in the Information and Telecommunications sector (ICT), the Communications and Information Technology Commission (CITC) has developed a cybersecurity maturity framework to regulate the best practices. The framework is called the Cybersecurity Regulatory Framework (CRF). It was built originally to be used by licensed service providers in Saudi Arabia to fulfill the minimum security requirements. However, CRF is comprehensive in a way that makes it applicable in other types of organizations as well. CRF is developed to regulate the cybersecurity domain and increases its maturity. Moreover, it helps establish best practices in information security in the ICT sector by defining different cybersecurity requirements. Additionally, CRF ensures that the organization’s services have confidentiality, integrity, and availability (CIA). This framework is based on different international standards, such as ISO/IEC 27001, NIST, KSA NCA Essential Cybersecurity Controls (more details were provided in the previous section). Moreover, CRF follows the Saudi laws in E-Transaction and E-Crimes.

**Figure 3:**
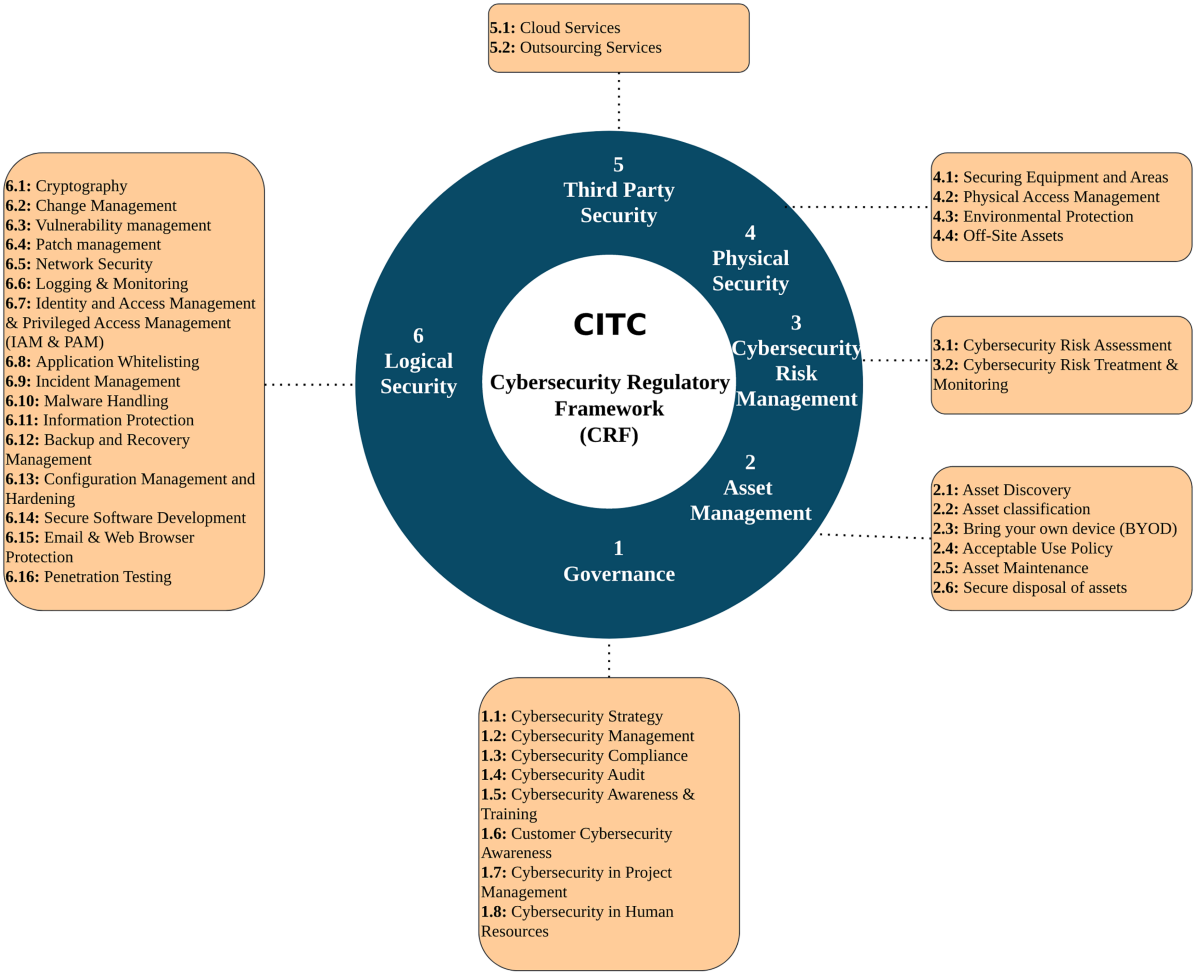
Cybersecurity Regulatory Framework (CRF).

As shown in Fig. 3, CRF requirements are categorized into six domains: Governance, Asset Management, Cybersecurity Risk Management, Logical Security, Physical Security, and Third-Party Security. CRF incorporates three compliance levels: basic, advanced, and efficient monitoring & continuous improvement. The purpose of the last level is to monitor the efficiency of the basic and advanced security controls. The requirements are categorized by different compliance levels using a risk-based method. To comply with a CRF high level, there should be compliance with all the preceding levels (Communications and Information Technology Commission of Saudi Arabia (CITC), 2019).

As can be concluded from the above Saudi frameworks, CRF is a general cybersecurity regulatory framework, whereas ECC provides security controls for any ITC based system. Firstly, we could not find any existing research studies that highlight these two standards and define the relationship between them. Moreover, There is no clear, published, practical assessment approach that can be followed to ensure continuous compliance with ECC and CRF and to measure the security level of the HEI IT systems.

### Holistic cybersecurity maturity assessment framework (HCYMAF)

**Figure 4:**
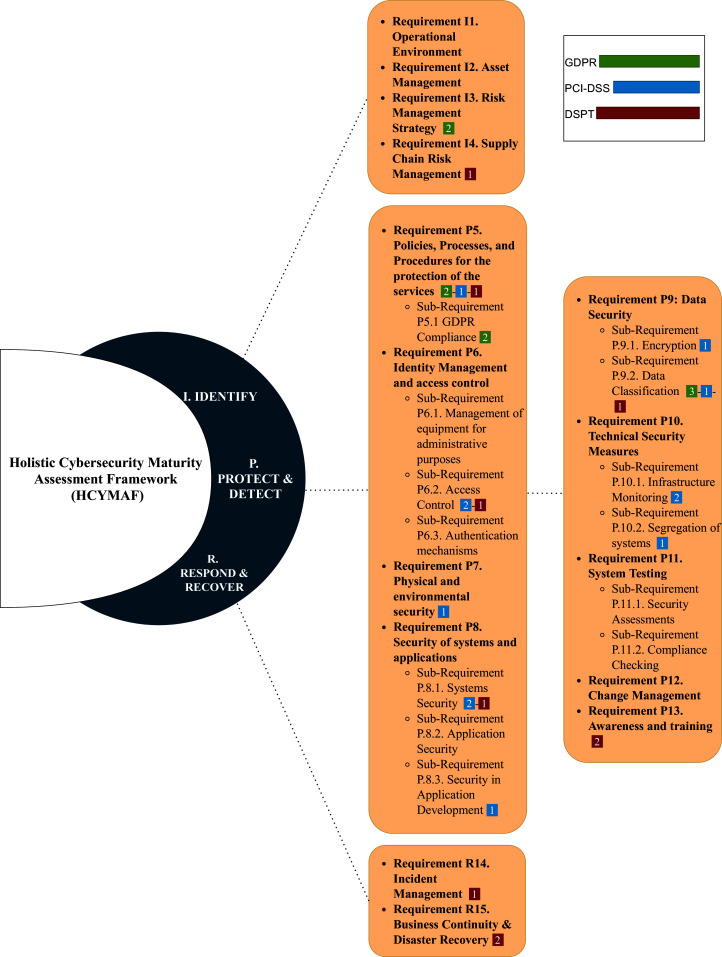
Cybersecurity Maturity Assessment Framework (HCYMAF).

Aliyu et al. (2020) presented a maturity model called Holistic Cybersecurity Maturity Assessment Framework (HCYMAF) for HEIs in the United Kingdom (UK). This model aims to measure the cybersecurity maturity of an organization by comparing it to the security best practices. Additionally, HCYMAF can serve as a gap-analysis and compliance-checking tool. The framework was built by reviewing the security requirements applicable to HEIs and with compliance to UK-recognized standards and regulations, including General Data Protection Regulation (GDPR) (EU, 2016), Payment Card Industry Data Security Standard (PCI DSS) (PCI Security Standards Council, 2018), and Data Security and Protection Toolkit (DSPT) (NHS Digital, 2020). HCYMAF has considered 15 general security requirements along with their sub-requirements. These requirements were selected based on various controls and standards, such as NIST framework (Barrett, 2018) and the Center for Internet Security (CIS) controls (Keller, 2019), and then were refined to be used for HEIs. These requirements were categorized into three groups: IDENTIFY (I), PROTECT & DETECT (P), and RESPOND & RECOVER (R) as shown in Fig. 4.

IDENTIFY requirements are important to understand the institution business and its operational ecosystem. For PROTECT & DETECT requirements, they are essential to detect different types of incidents and provide all kinds of protection to the institution’s services and assets. RESPOND & RECOVER requirements are necessary to respond properly when an incident happens and to recover after the attack. The division of the requirements is as follows:

- IDENTIFY requirements are from I1 to I4.
- PROTECT & DETECT requirements are from P5 to P13.
- RESPOND & RECOVER requirements are from R14 to R15.

The chosen regulations and standards (GDPR, PCI DSS, and DSPT) are then mapped with the HCYMAF requirements. Figure 4 also highlights this mapping. The numbers next to each requirement indicate the number of the standard’s requirements mapped to each HCMAF requirement. For example, requirement P5 in HCYMAF is mapped to two requirements in GDPR, one requirement in PCI-DSS, and one requirement in DSPT.

Moreover, the HCYMAF model consists of six maturity levels, ranges between 0 (the lowest) and five (the highest). To evaluate the framework and prove its effectiveness, interviews with security experts and a case study were conducted. Also, the model was validated by getting feedback from the scientific communities that were in charge of reviewing their academic articles related to HCYMAF model.

### SCMAF security requirements and mappings

As can be concluded from work done in assessing the cybersecurity maturity level in SA organizations in general and HEIs in particular, there is a need to build a SA Cybersecurity Maturity Assessment Framework for HEIs (SCMAF). This framework needs to be comprehensive by considering the international standards and customized to fulfill the SA local cybersecurity regulatory frameworks. Also, this framework should offer a lightweight, automated assessment process to the HEIs so they can self-assess their security maturity level in a convenient and confidential way.

Therefore, to build SCMAF, all the requirements and security controls defined in CITC’s CRF and NCA’s ECC by the governmental agencies in Saudi Arabia were collected and studied. Then, a recent international cybersecurity maturity assessment model specialized for HEI was selected, which is HCYMAF that is introduced by Aliyu et al. (2020). Table 2 defines the list of SCMAF requirements and show their mappings with all related frameworks, HCYMAF, ECC, and CRF.

**Table 2:** Saudi cybersecurity maturity assessment framework requirements and mappings.

	SCMAF Requirement	Standards Mapping
IDENTIFY	SCMAF I1: The organization and people responsible shall define the cybersecurity strategy and compliance with security standards laws. All the security requirements applicable shall be defined.	HCYMAF: I1
		ECC: 1–1, 1–2, 1–3, 1–4
		CRF: 1.1, 1.2, 1.3
	SCMAF I2: The organization shall identify and record all assets (organizational and personal assets) and dispose unused assets securely.	HCYMAF: I2
		ECC: 2–1, 2–14
		CRF: 2.1, 2.2, 2.4, 2.5, 2.6
	SCMAF I3: The organization shall implement risk management and assessment strategy.	HCYMAF: I3
		ECC: 1–5
		CRF: 3.1, 3.2
	SCMAF I4: The organization shall ensure the use of third-party services securely.	HCYMAF: I4
		ECC: 4–1, 4–2
		CRF: 6.1, 6.2
DETECT & PROTECT	SCMAF P5: The organization shall ensure the protection of the services.	HCYMAF: P5
		ECC: 2–5, 2–7
		CRF: 4.5, 4.11
	SCMAF P6.1: The organization shall ensure the physical equipment security.	HCYMAF: P6.1
		ECC: 2–2, 2–14
		CRF: 4.7, 5.2
	SCMAF P6.2: The organization shall manage the access to privileged resources.	HCYMAF: P6.2
		ECC: 2–2
		CRF: 4.7
	SCMAF P6.3: The organization shall define and identify the authentication and access control mechanisms.	HCYMAF: P6.3
		ECC: 2–2
		CRF: 4.7
	SCMAF P7: The organization shall ensure the security of the environment for the physical equipment.	HCYMAF: P7
		ECC: 1–5, 2–14, 3–1
		CRF: 3.1, 5.1, 5.3, 5.4
	SCMAF P8.1: The organization shall ensure the security of its systems.	HCYMAF: P8.1
		ECC: 2–10, 1–6
		CRF: 4.3, 4.8, 4.14
	SCMAF P8.2: The organization shall ensure the security of its applications.	HCYMAF: P8.2
		ECC: 2–10, 1–6
		CRF: 4.3, 4.8, 4.14
	SCMAF P8.3: The organization shall follow the best practices to develop applications securely.	HCYMAF: P8.3
		ECC: 1–6, 2–15
		CRF: 1.7, 4.14
	SCMAF P9.1: The organization shall encrypt sensitive information.	HCYMAF: P9.1
		ECC: 1–5, 2–8, 2–5, 2–7
		CRF: 3.1, 4.1, 4.5, 4.11
	SCMAF P9.2: The organization shall ensure all the data are classified.	HCYMAF: P9.2
		ECC: 1–5, 2–8
		CRF: 3.1, 4.1
	SCMAF P10: The organization shall ensure the security of network services.	HCYMAF: P10
		ECC: 2–3, 2–10, 2–5, 2–13, 2–4, 2–11
		CRF: 4.4, 4.5, 4.9, 4.10, 4.16
	SCMAF P10.2: The organization shall segregate the system based on the risk.	HCYMAF: P10.2
		ECC: 1–5, 2–5, 2–2, 1–6, 4–2
		CRF: 3.1, 4.5, 4.7, 4.14, 6.1
	SCMAF P11.1: The organization shall do periodic security assessments.	HCYMAF: P11.1
		ECC: 1–8, 4–1, 4–2
		CRF: 1.4, 6.1, 6.2
	SCMAF P11.2: The organization shall regularly check the compliance of regulation and standards recognized in KSA.	HCYMAF: P11.2
		ECC: 1–7, 1–8, 1–6, 4–1
		CRF: 1.3, 1.4, 4.14, 6.2
	SCMAF P12: The organization shall maintain and manage the changes.	HCYMAF: P12
		ECC: 1–1, 1–6, 1–5, 4–1, 4–2
		CRF: 1.1, 4.2, 3.1, 6.1, 6.2
	SCMAF P13: The organization shall ensure the employees are well trained to face security threats.	HCYMAF: P13
		ECC: 1–9, 1–10, 2–13
		CRF: 1.5, 1.6, 1.8, 4.9
	SCMAF P14: The organization shall ensure the protection of emails and web browsers.	HCYMAF: N/A
		ECC: 2–4
		CRF: 4.15
RESPOND & RECOVER	SCMAF R15: The organization shall manage and define strategies for handling incidents.	HCYMAF: R14
		ECC: 2–13, 3–1
		CRF: 4.9
	SCMAF R16: The organization shall ensure the continuity of services and do backup for a fast recovery.	HCYMAF: R15
		ECC: 2–1, 2–9
		CRF: 2.5, 4.12

We followed the same categorization of the security requirements in HCYMAF, which are "IDENTIFY", "PROTECT & DETECT", and "RESPOND & RECOVER". For example, the first requirement in the proposed framework "SCMAF I1" under IDENTIFY category was mapped to HCYMAF I1, ECC 1–1, 1–2, 1–3, 1–4, and CRF 1.1, 1.2, 1.3. The requirements of the three existing standards and their identifiers were highlighted in the above two subsections.

**Figure 5:**
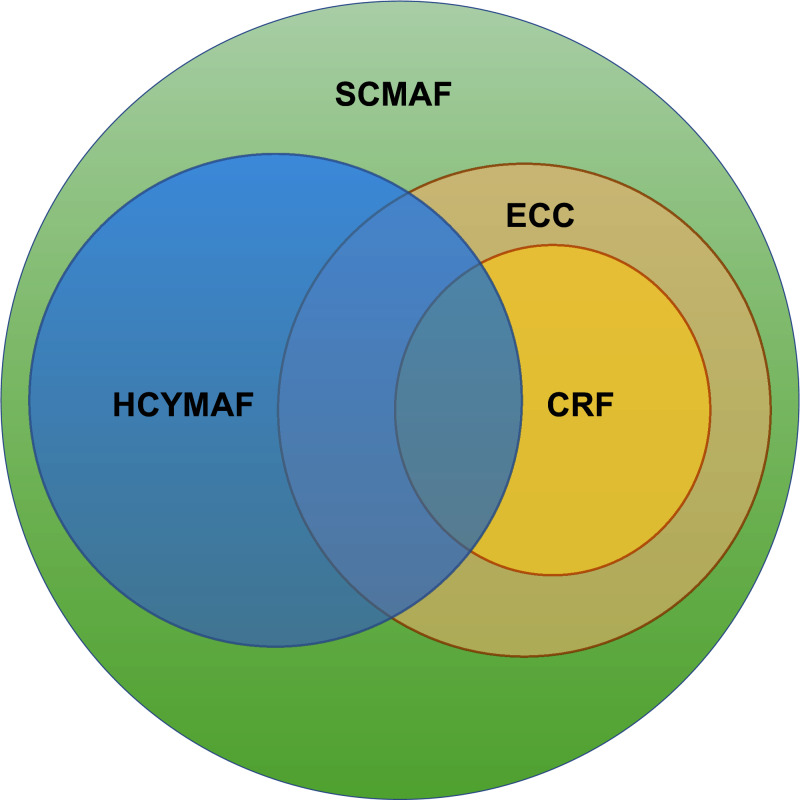
Saudi Cybersecurity Maturity Assessment Framework (SCMAF).

The mapping results reveal that almost all CRF requirements were included in ECC. Also, HCYMAF and SA standards have much in common. Figure 5 illustrates the overlapping among the three frameworks and how the proposed SCMAF is comprehensive enough to (a) include all the requirements defined by them, (b) add missing security requirements, and (3) exclude the requirements that are not applicable in the context of higher education.

For example, one of the requirements (SCMAF P14) is added by the proposed SCMAF, inspired by CRF, but is missing in HCYMAF. SCMAF P14 is related to utilizing standardized security mechanisms for email and web browser protection. This is an essential requirement that needs to be considered and assessed in HEIs as these two services are highly demanded and used in the education sector.

On the other hand, there are requirements in CRF and ECC that do not apply to HEIs, such as those related to industrial control systems. In this case, they were considered not applicable and were excluded from the framework’s requirements. The complete list of SCMAF requirements and their descriptions in addition to the entire mapping with the SA and international security frameworks are shown in Table 2.

### The implementation of the proposed SCMAF framework

To implement the proposed SCMAF and illustrate how it could be applied to assess the HEI systems and measure their cybersecurity maturity levels, a demonstration study is presented in this section. This study provides an exercise that (a) goes through the general flow of the proposed framework, (b) examines the level of maturity for each requirement by raising well-designed questions that address the degree of fulfilling the requirement, and (c) calculates an overall score and attaches it with a summary report.

**Figure 6:**
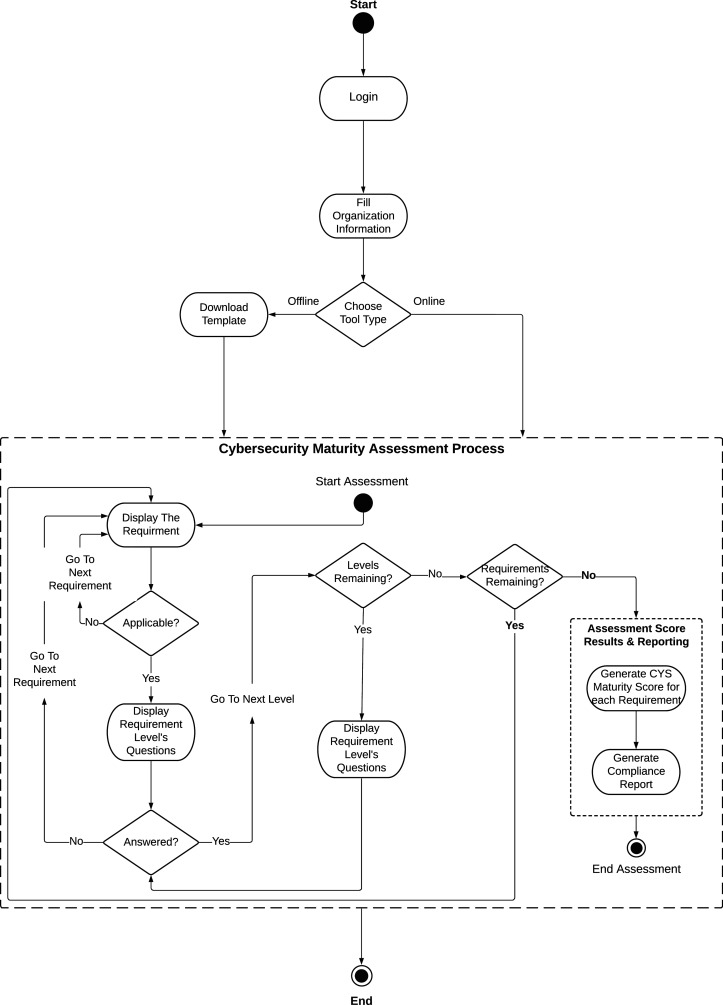
Saudi Cybersecuirty Maturity Assessment Framework (SCMAF) system flow.

Figure 6 shows the SCMAF system flow. As mentioned before, SCMAF is offered to the HEIs in terms of a lightweight automated tool that can be used online or downloaded to be executed offline. To get a copy of the assessment tool, the institution has to register with the tool’s provider and fill its information in order to login and access the tool. Then, based on the choice of the institution, the online or the offline version of the tool can be executed.

The SCMAF requirements are measured one by one. If the requirement is applicable by the institution, the related questions of the first level will be displayed. If these questions are answered with yes, then the following level’s questions will appear, and so forth. The level’s questions won’t be shown unless all preceding levels within the same requirement are achieved. Once the levels per requirement are examined, the tool moves to the following requirement and repeats the same process. The system follows client-server architecture with approximate computational complexity of O(n*k), where *n* is the number of requirements and *k* is the number of questions per requirement. The complexity was computed by considering all steps in the flow diagram to run in a constant time except going through the requirements and their corresponding questions.Thus, these steps will cost n*k operations, based on the number of requirements and questions.

A total of six levels of maturity are considered by SCMAF similar to HCYMAF model. The levels are summarized as follows:

- **Level 0**: INCOMPLETE. The requirement is either unknown, not applicable, *ad hoc*, or may not get completed.
- **Level 1**: INITIAL. The requirement is reactive and unpredictable. It could be achieved but with delay and over budget.
- **Level 2**: MANAGED. The achievement of the requirement is well planned, implemented, measured and under control.
- **Level 3**: DEFINED. The institution process to achieve the requirement is proactive, standardized and well-guided.
- **Level 4**: QUANTITATIVELY MANAGED. The institution is data-driven with quantitative performance indicators to streamline with the internal and external stakeholders’ needs.
- **Level 5**: OPTIMIZED. The institution approves a continuous improvement strategy for the requirement to ensure well adaptation to future changes.

**Figure 7:**
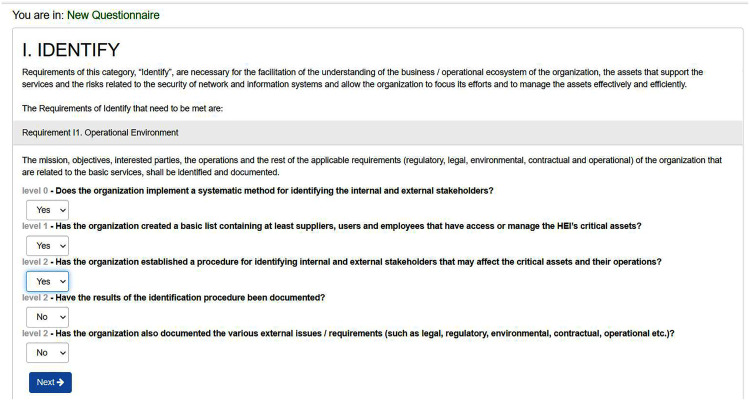
Requirement level’s questions sample.

The description of each level for each requirement is displayed to the institution to answer whether this level is achieved by the institution or not. The institution has only to reply by ‘yes’ to the level it is achieving per requirement. Otherwise, it replies with ‘no’ as shown in Fig. 7. Table 3 shows the levels of requirements that might be provided by an institution based on its status. For example, SCMAF I1 was not applicable by the institution, so it selected level 0 for it. Whereas, SCMAF R15, SCMAF R16, SCMAF P11.1, SCMAF I2, and SCMAF 14 are given levels of security 1–5 respectively.

**Table 3:** Saudi cybersecurity maturity assessment framework-demo study.

Requirement	Level	Requirement	Level	Requirement	Level
SCMAF I1	0	SCMAF P7	2	SCMAF P11.1	3
SCMAF I2	4	SCMAF P8.1	1	SCMAF P11.2	0
SCMAF 13	2	SCMAF P8.2	4	SCMAF P12	5
SCMAF 14	5	SCMAF P8.3	5	SCMAF P13	2
SCMAF P5	2	SCMAF P9.1	1	SCMAF P14	1
SCMAF P6.1	1	SCMAF P9.2	5	SCMAF R15	1
SCMAF P6.2	2	SCMAF P10	1	SCMAF R16	2
SCMAF P6.3	5	SCMAF P10.2	1		

**Figure 8:**
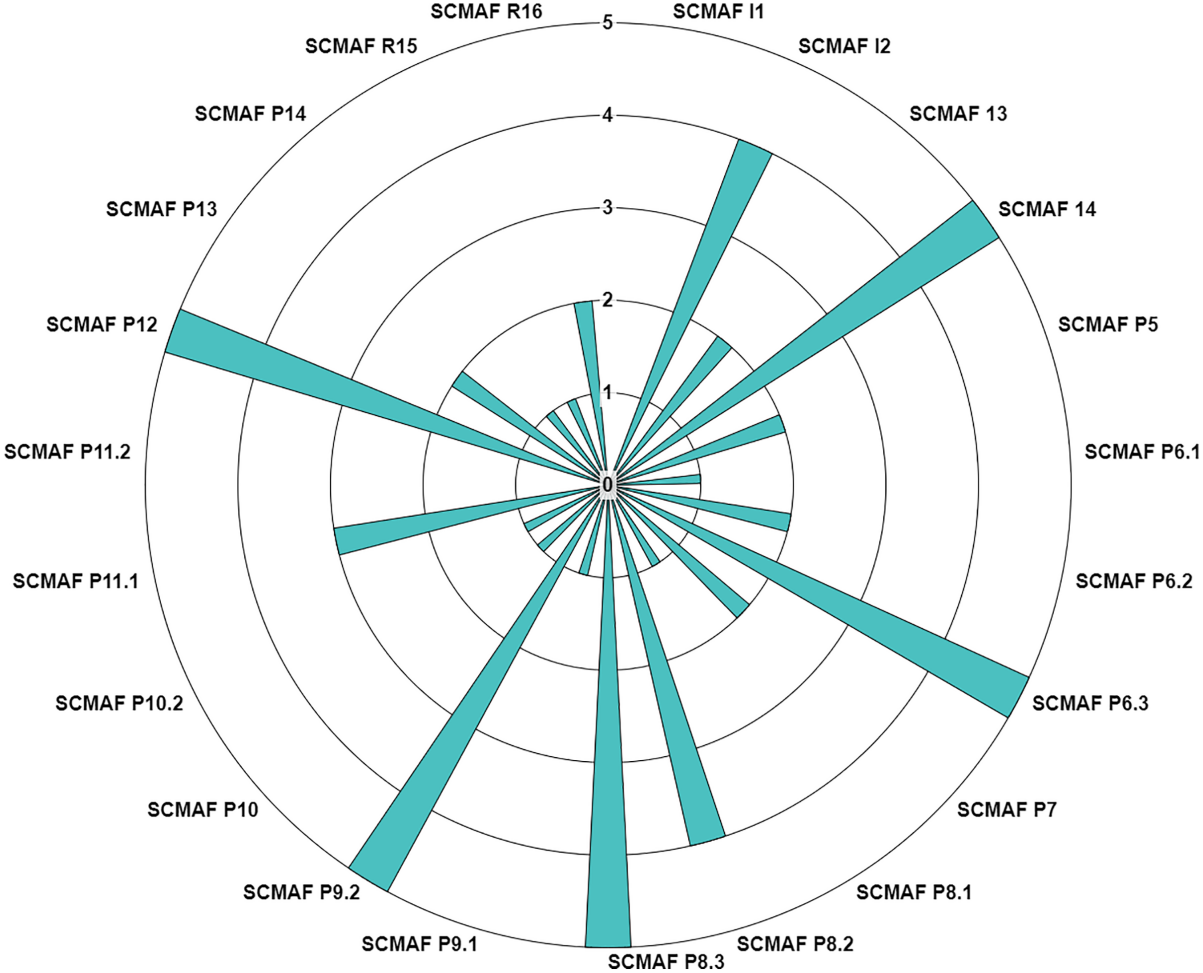
SCMAF Requirements’ levels chart.

Figure 8 illustrates how the institution observes the level of each requirement after their inputs by answering the questions. So, by only checking the chart, the institution can conclude the maturity level achieved by each requirement. Therefore, in this demo study, the institution reached the following levels for its requirements:

- **INCOMPLETE**: SCMAF I1, and SCMAF P11.2.
- **INITIAL**: SCMAF P6.1, SCMAF P8.1, SCMAF P9.1, SCMAF P10, SCMAF P10.2, SCMAF P14, and SCMAF R15.
- **MANAGED**: SCMAF P5, SCMAF P6.3, SCMAF P7, SCMAF P13, and SCMAF R16.
- **DEFINED**: SCMAF P11.1.
- **QUANTITATIVELY MANAGED**: SCMAF I2, and SCMAF P8.2.
- **OPTIMIZED**: SCMAF I4, SCMAF P6.3, SCMAF P8.3, SCMAF P9.2, and SCMAF P12.

**Figure 9:**
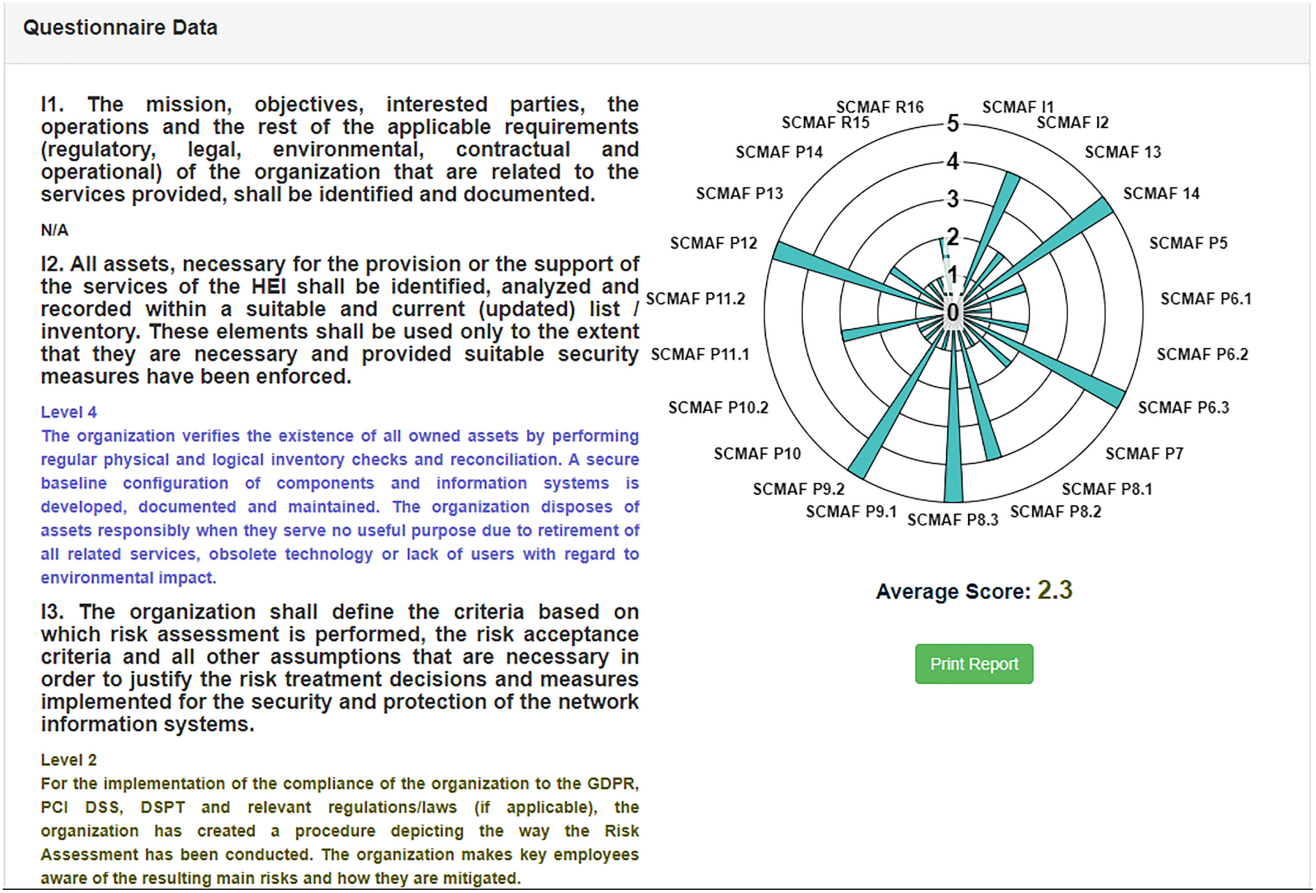
Saudi Cybersecurity Maturity Assessment Framework (SCMAF)-report screenshot.

After all requirements are assessed, the overall cybersecurity maturity score of the institution is calculated. Figure 9 provides a screenshot from the tool where it shows (a) the final score achieved by the institution, (b) the requirements levels chart, and (c) a summary of each requirement and which level it secures. From this screenshot, we can see that SCMAF I1 is not applicable. SCMAF I2 achieves level 4 where a description of level 4 is provided, SCMAF I3 reaches level 2, and also level 2 description is provided. This applies to the rest of the requirements. The institution can download and print the full report as well.

Based on the assessment results, the institutions become aware of their shortcomings regarding cybersecurity and start preparing enhancement plans according to their priorities.

## Conclusions

This research has proposed SCMAF, a comprehensive customized cybersecurity maturity assessment framework for HEI in Saudi Arabia (SA). The framework has considered international cybersecurity standards in addition to SA cybersecurity regulatory frameworks. This framework is offered to the institutions to apply self-assessment of their IT-based systems to measure their security levels. Consequently, addressing their weaknesses and preparing mitigation plans and continuous improvement.

To build a specialized framework for SA HEIs, we studied the current cybersecurity standards approved and followed in SA. Additionally, we investigated and utilized recent international cybersecurity maturity models. Therefore, SA CITC’s CRF and NCA’s ECC are deeply analyzed and mapped in addition to the international maturity model, HCYMAF. All the defined SCMAF requirements are mapped to these models’ requirements and security controls to introduce a comprehensive cybersecurity maturity framework dedicated to SA HEIs. SCMAF has defined 16 main requirements in addition to the sub-requirements. The achievement of these requirements by the institution is ranged among six different maturity models: INCOMPLETE, INITIAL, MANAGED, DEFINED, QUANTITATIVELY MANAGED, and OPTIMIZED. Each maturity level in each requirement has a clear, well-designed description displayed to the institutions where they need to confirm if they apply it or not.

After assessing all requirements, a total score representing the cybersecurity maturity of the institution’s systems will be calculated. This score is communicated to the institution in terms of a visual chart and a detailed report. The assessment framework proposed by SCMAF is shared with the HEIs as an online web-based tool or as an offline tool that needs to be downloaded on the institution’s premises. The institution can choose the version that suits their interest and confidentiality. Applying regular assessments for the IT-based systems in any institution and specific in HEI is essential to maintain the functionality of their services and protect them from being threatened by security attacks. Moreover, the framework should always be up-to-date to adapt with the new security requirements, attacks, and mechanisms. The main SCMAF contribution is to offer a comprehensive, user-friendly, up-to-date, and continuous cybersecurity assessment process to HEIs in Saudi Arabia.

## Limitations and future works

Even though the proposed SCMAF framework covers many aspects that help assess the organizations’ cybersecurity maturity, it can be improved by allowing the organizations to upload evidence to prove that they have met the security requirement(s). Also, the tool can be customized to meet different organization’s needs.

For future work, the framework can be enhanced to be accurately mapped with critical international standards like ISO2700 as a preliminary step for institutions to becoming cybersecurity-certified. Moreover, the compliance report can be customized based on the institution’s needs.

Finally, SCMAF can also be adapted to be applied to different sectors other than education, such as healthcare and industrial organizations.

## Additional Information and Declarations

### Data Availability

The following information was supplied regarding data availability: The article presents a novel cybersecurity assessment framework. There are no raw data or code; all the necessary information appears in the article.
